# Supplementary material for: QSAR Models for Human Carcinogenicity: An Assessment Based on Oral and Inhalation Slope Factors
Source: Molecules. 2020 Dec 29;26(1):127. doi: 10.3390/molecules26010127 (PMC7796303; doi:10.3390/molecules26010127)

**Supplementary Materials of QSAR models for the human carcinogenicity: an assessment based on oral and inhalation slope factors**

Cosimo Toma, Alberto Manganaro, Giuseppa Raitano, Marco Marzo, Domenico Gadaleta, Diego Baderna, Alessandra Roncaglioni, Nynke Kramer, Emilio Benfenati.

**Table S1. Non-exhaustive list of software tools to predict carcinogenicity, adapted from Bossa et al., 2018 [32].** C: commercial software, F: freely available, S: statistical, A: alert-based models, K: knowledge-based models.

| Software | Availability | Model category |
| --- | --- | --- |
| ACD/Percepta Impurities Suite | C | S |
| AMBIT (IdeaConsult Ltd.) | F | S, A, K |
| Danish QSAR Predictions database (DK EPA) | F | S, A, K |
| DEREK Nexus Lhasa Ltd. | C | K |
| HazardExpert Pro and ToxAlert (CompuDrug Ltd) | C | A |
| LAZAR (*in silico* toxicology, GmbH) | F | S |
| Leadscope Model Applier (Leadscope, Inc.) | C | S, A |
| QSAR (Molcode Ltd) | C | S |
| Multicase CASE Ultra Models (MultiCASE Inc.) | C | S, K |
| OECD QSAR Toolbox (OECD) | F | K |
| Oncologic (US EPA) | F | K |
| PASS (Institute of Biomedical Chemistry of the Russian Academy of Medical Sciences, Moscow) | C | S |
| Toxtree (EU JRC—Ideaconsult Ltd.) | F | A, K |
| VEGA (Istituto di Ricerche Farmacologiche Mario Negri) | F | S, A |

**Table S2. Descriptors of the OSF and ISF models. *OSF_class*** *and* ***ISF_class*** *stand respectively for Oral and Inhalation slope factor classification models as implemented in VEGA,* ***OSF_reg*** *and* ***ISF_reg*** *stand respectively for Oral and Inhalation slope factor regression models as implemented in KNIME,* ***OSF_reg_VEGA*** *and* ***ISF_reg_VEGA*** *stand respectively for Oral and Inhalation slope factor regression models as implemented in VEGA.*

| **Endpoint** | **Split scheme** | **No. of descriptors** | **Descriptors** |
| --- | --- | --- | --- |
| OSF_class | A | 7 | nS, nCIC, ATSC6s, P_VSA_logp_6, SpMax_EA(dm), B02[C-N], B09[C-F] |
| ISF_class | B | 9 | piPC10, ATSC2p, EEig15(bo), F01[N-N], BEL3m, GATS1v, PW4, MATS3s, BEH4e |
| OSF_reg | A | 12 | nCIC, GATS2s, P_VSA_m_5, SM08_EA(bo), nRNNOx, nFuranes, N-067, CATS2D_03_DA, CATS2D_04_AL, B05[O-O], B08[Cl-Cl], F04[O-Cl] |
|  | B | 12 | nC, nN, J_Dz(p), SpDiam_AEA(dm), Eig14_EA(dm), CATS2D_03_DA, B02[C-C], B02[C-O], B04[O-Cl], B08[C-N], B08[Cl-Cl], F04[C-C] |
| ISF_reg | A | 10 | nN, X4v, MATS4m, MATS6i, Eig11_AEA(bo), nRNNOx, N-067, B04[O-Cl], B08[Cl-Cl], F06[Cl-Cl] |
|  | B | 12 | RFD, VE1sign_B(m), ATSC4e, MATS6i, GGI6, nRNNOx, N-067, B02[Cl-Cl], F04[C-O], F04[O-Cl], F08[C-S], DLS_05 |
| OSF_reg_VEGA | N/A | 12 | nCIC, GATS2s, P_VSA_m_5, SM08_EA(bo), nRNNOx, nFuranes, N-067, CATS2D_03_DA, CATS2D_04_AL, B05[O-O], B08[Cl-Cl], F04[O-Cl] |
| ISF_reg_VEGA | N/A | 12 | C-041, ATS8m, GATS6p, CATS2D_03_DL, CATS2D_07_DL, nN-N, IC4, B02[Cl-Cl], B04[O-Cl], B07[Cl-Cl], B08[Cl-Cl], SRW7 |

**Table S3. Sizes of datasets for the regression model.** For the two endpoints, the size of the data sets obtained applying 1) the two-split and 2) the single split scheme is reported.

|  | REGRESSION | | | | CLASSIFICATION | |
| --- | --- | --- | --- | --- | --- | --- |
| Endpoint | Splitting | Training set | Test set | External | Training set | Test set |
|  | scheme |  |  | validation set |  |  |
| OSF | A | 226 | 57 | 32 | 593 | 149 |
|  | B | 252 | 63 | - |  |  |
| ISF | A | 188 | 48 | 27 | 598 | 151 |
|  | B | 210 | 53 | - |  |  |

**Table S4. QSAR Model Reporting Format of the carcinogenicity.** For each model, we listed the link to the relative QMRF on our platform.

| **Model** | **Link** |
| --- | --- |
| Oral slope factor – Classification model | https://www.vegahub.eu/vegahub-dwn/qmrf/QMRF_SFO_CLASS.pdf |
| Oral slope factor – Regression model | https://www.vegahub.eu/vegahub-dwn/qmrf/QMRF_SFO_REG.pdf |
| Inhalation slope factor – Classification model | https://www.vegahub.eu/vegahub-dwn/qmrf/QMRF_SFI_CLASS.pdf |
| Inhalation slope factor – Regression model | https://www.vegahub.eu/vegahub-dwn/qmrf/QMRF_SFI_REG.pdf |

**Figure S1 Inhalation CART model**


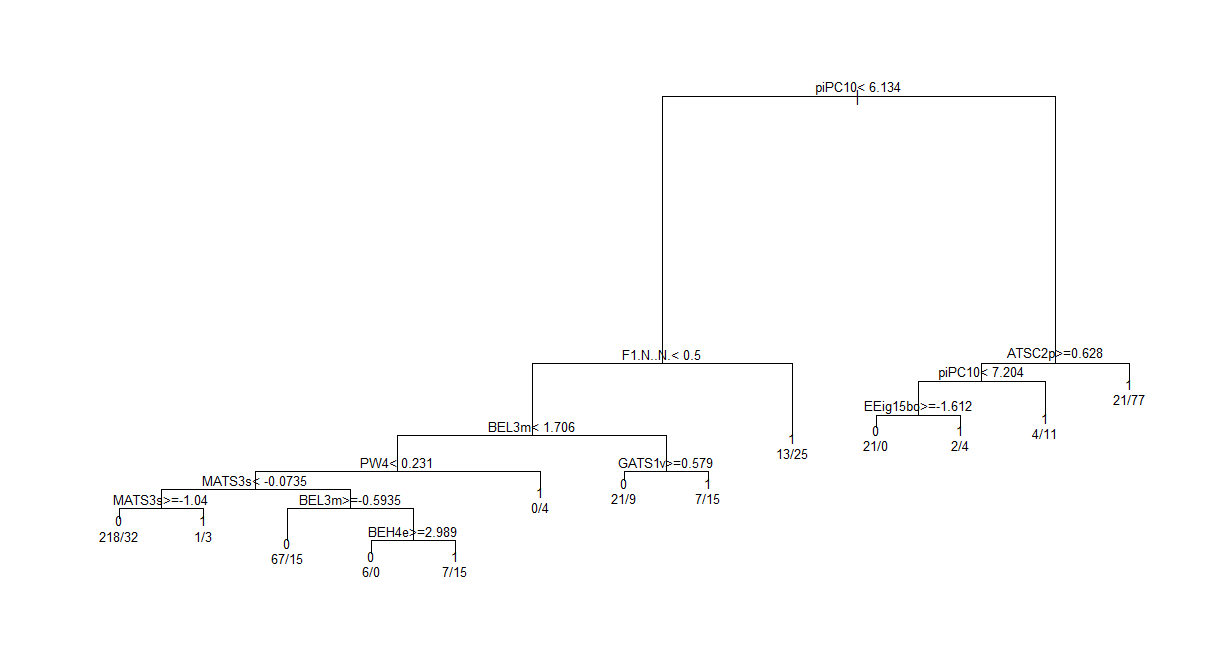


**Figure S2 Oral CART model**


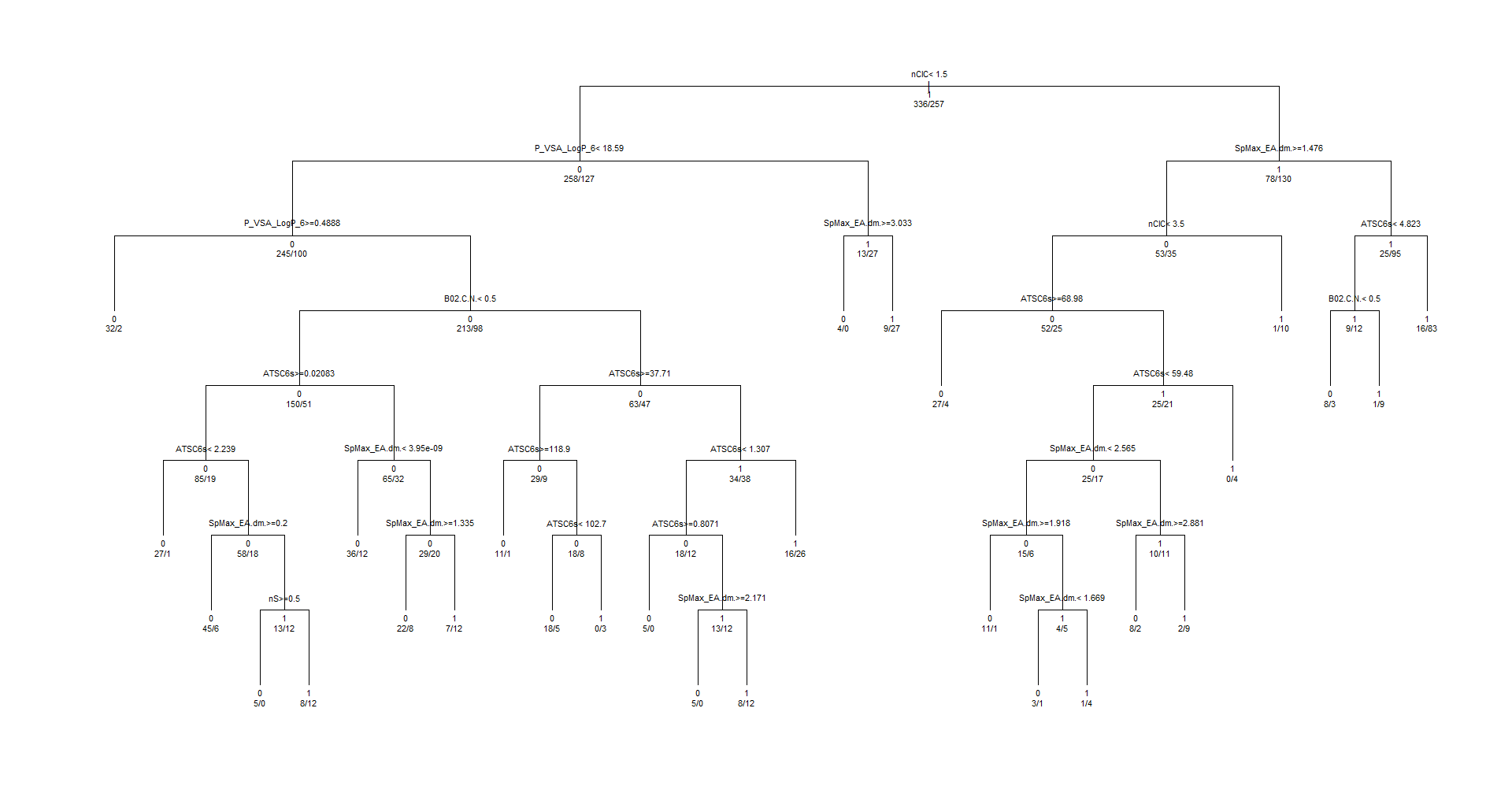

Supplement: Supplementary file 1 [file molecules-26-00127-s001.zip › Supplementary Materials_rev.docx]
